# Immunity

**DOI:** 10.3201/eid2204.151858

**Published:** 2016-04

**Authors:** Theodore M. Lee

**Affiliations:** Peachtree Allergy and Asthma Clinic, PC, and Emory University School of Medicine, Atlanta, Georgia, USA

**Keywords:** HIV/AIDS and other retroviruses, allergy and immunology, interleukin-4, lymphocytes, book review, history

Immunity, written by William E. Paul, MD, is a unique synthesis of autobiography and perspective on the history of immunology with easy-to-follow and informative explanations of key concepts. The author’s death occurred in September 2015, just before publication of this book, his final manuscript. His discovery of interleukin-4 and his experiences as head of the Office of AIDS Research at the National Institutes of Health (Bethesda, MD, USA) are among the author’s many outstanding achievements chronicled in the book. One chapter is devoted to Dr. Paul’s previously described concept of the laws of immunology: universality, the idea that cells and products of the immune system are capable of recognizing an unlimited array of substances; tolerance, the notion that the response to self-antigens is controlled or eliminated; and appropriateness, the concept that the immune response is appropriate to the inducing pathogen. 

Several figures in the book provide additional insights into this publication’s distinctiveness: a photograph of one of Dr. Paul’s mentors, Baruj Benacerraf, receiving the Nobel Prize; another photograph of a German 200-mark note featuring Paul Ehrlich, a Nobel Prize winner for his work in immunology; a photograph showing an Austrian 1,000-schilling note with the image of Karl Landsteiner, Nobel Prize winner for discovery of the immunology of blood groups; a diagram illustrating a peptide bound into the groove of a class I major histocompatibility complex molecule; and an electron microphotograph of a dendritic cell with adherent lymphocytes. The author was a major participant in remarkable advances in our understanding of immunology. For example, he states, “My professor of microbiology… was deeply skeptical about the function of lymphocytes…. Lymphocytes are so unprepossessing in appearance that many believed their chief function was to serve as nutrients for cells that were thought to be more important in antimicrobial responses….” Dr. Paul provides details of his discovery of the molecular structure and functions of interleukin-4, a critical regulatory factor for lymphocytes.

Furthermore, Dr. Paul offers insights on recent developments, such as the hygiene hypothesis of the genesis of allergic disease; inflammasomes; colony collapse disorder in honeybees; and clinical trials with dupilumab. He discusses these issues with the same profundity and clarity that he provides on his work in the frontiers of immunologic research early in his distinguished career. His response to the AIDS crisis provides an inspiring perspective for public health officials confronted with urgent challenges. He states, “In the end, I concluded that I had to take on the responsibility. The AIDS epidemic was such that one really didn’t have a choice.... I was determined that while the planning process had to represent the best thinking in the field, it could not be too proscriptive. We didn’t want to quash creativity, without which progress was sure to be hampered.” Dr. Paul is recognized as being an important catalyst in the development of effective antiviral therapy and has been called a hero of the AIDS epidemic. 

Few readers will not be impressed by Dr. Paul’s energy, enthusiasm, and innovative spirit. This book will be relished by those who are captivated by the history of modern biologic and medical science.

